# Prognostic relevance of the revised R status definition in pancreatic cancer: meta-analysis

**DOI:** 10.1093/bjsopen/zrac010

**Published:** 2022-03-18

**Authors:** Carl Stephan Leonhardt, Willem Niesen, Eva Kalkum, Rosa Klotz, Thomas Hank, Markus Wolfgang Büchler, Oliver Strobel, Pascal Probst

**Affiliations:** Department of General, Visceral and Transplantation Surgery, University of Heidelberg, Heidelberg, Germany; Department of General Surgery, Division of Visceral Surgery, Medical University of Vienna, Vienna, Austria; Department of General, Visceral and Transplantation Surgery, University of Heidelberg, Heidelberg, Germany; Study Center of the German Society of Surgery, University of Heidelberg, Heidelberg, Germany; Department of General, Visceral and Transplantation Surgery, University of Heidelberg, Heidelberg, Germany; Study Center of the German Society of Surgery, University of Heidelberg, Heidelberg, Germany; Department of General, Visceral and Transplantation Surgery, University of Heidelberg, Heidelberg, Germany; Department of General Surgery, Division of Visceral Surgery, Medical University of Vienna, Vienna, Austria; Department of General, Visceral and Transplantation Surgery, University of Heidelberg, Heidelberg, Germany; Department of General, Visceral and Transplantation Surgery, University of Heidelberg, Heidelberg, Germany; Department of General Surgery, Division of Visceral Surgery, Medical University of Vienna, Vienna, Austria; Department of General, Visceral and Transplantation Surgery, University of Heidelberg, Heidelberg, Germany; Study Center of the German Society of Surgery, University of Heidelberg, Heidelberg, Germany; Department of Surgery, Cantonal Hospital Thurgau, Frauenfeld, Switzerland

## Abstract

**Background:**

The prognostic impact of margin status is reported with conflicting results after pancreatic cancer resection. While some studies validated an uninvolved resection margin (R0) 1 mm or more of tumour clearance, others have failed to show benefit. This systematic review and meta-analysis aimed to investigate the effects of margin definitions on median overall survival (OS).

**Methods:**

MEDLINE, Web of Science, and the Cochrane Central Register of Controlled Trials were searched for studies reporting associations between resection margins and OS between 2010 and 2021. Data regarding margin status (R0 circumferential resection margin (CRM) negative (CRM–), R0 CRM positive (CRM+), R0 direct, and R1 and OS were extracted. Hazard ratios (HRs) were pooled with a random-effects model. The risk of bias was evaluated with the Quality in Prognosis Studies (QUIPS) tool.

**Results:**

The full texts of 774 studies were screened. In total, 21 studies compromising 6056 patients were included in the final synthesis. In total, 188 (24 per cent) studies were excluded due to missing margin definitions. The R0 (CRM+) rate was 50 per cent (95 per cent confidence interval (c.i.) 0.40 to 0.61) and the R0 (CRM−) rate was 38 per cent (95 per cent c.i. 0.29 to 0.47). R0 (CRM−) resection was independently associated with improved OS compared to combined R1 and R0 (CRM+; HR 1.36, 95 per cent c.i. 1.23 to 1.56).

**Conclusion:**

The revised R status was confirmed as an independent prognosticator compared to combined R0 (CRM+) and R1. The limited number of studies, non-standardized pathology protocols, and the varying number of margins assessed hamper comparability.

## Teaser

The prognostic significance of the revised R status in pancreatic cancer remains controversial. This meta-analysis and systematic review provides evidence that the revised R status serves as an independent prognosticator in upfront resected pancreatic cancer.

## Introduction

Pancreatic ductal adenocarcinoma (PDAC) is expected to become the second most common cause of cancer-related mortality in the USA by 2030^1^, but approximately 20 per cent of patients are candidates for surgical resection (currently the only potential cure) at the time of diagnosis. Resection margins in PDAC have been traditionally considered an indicator of surgical quality for adequate oncological resections. However, the frequency of local recurrences seems to be at odds with the reported rates of R0 resections, which vary widely between 10 to 80 per cent, due to differences in definitions of what is included in the term R0^2,3^. New protocols for pathological assessment of resection specimens and a revised definition for resection margins have been introduced. Initially introduced in 2002 by the UK Royal College of Pathologists, a wide resection margin with R0 of 1 mm or more from tumour cells to the margin was endorsed as a revised definition of R status by the International Study Group of Pancreatic Surgery, as well as the 8th edition of the AJCC Cancer Staging Manual^4–9^.

Although the importance of assessing circumferential resection margins (CRM) is now widely accepted, margins definition remains controversial^3^. The prognostic relevance of the revised R status^10^ has been confirmed in some studies, while others have failed to demonstrate such an association^11^. Similarly, recent meta-analyses have reported conflicting results, given the lack of strict criteria for margin definitions or the inclusion of patients treated with neoadjuvant therapy^12,13^. Owing to this heterogeneity, widespread adoption of the revised R0 definition is lacking, hampering comparability between studies and outcomes^10,14^.

This systematic review aimed to assess the prognostic role of the revised R status in patients with PDAC submitted to primary pancreatic resections undertaken with curative intent.

## Methods

This study is reported according to the PRISMA guidelines^15^.

### Systematic literature search

A systematic literature search was performed in the MEDLINE, Web of Science, and Cochrane Central Register of Controlled Trials (CENTRAL) databases on 14 January 2021^16^. The following search strategy was used for MEDLINE with the search strategies for the other databases available upon request:

(((‘resection margin’[tiab] OR ‘resection margins’[tiab] OR R1[tiab] OR R0[tiab] OR ((negative[tiab] OR positive[tiab]) AND margin*[tiab]) OR (prognostic[tiab] AND factor*[tiab]) OR prognosis[tiab] OR survival[tiab] OR (((lymph[tiab] AND node*[tiab]) OR nodal[tiab]) AND metastasis[tiab]) OR Prognosis [Mesh] OR ‘Margins of Excision’[Mesh]))) AND ((((pancreas[tiab] OR pancreatic) AND (cancer*[tiab] OR neoplasm*[tiab] OR carcinoma[tiab] OR tumor*[tiab] OR tumour*[tiab] OR adenocarcinoma[tiab])) OR PDAC[tiab] OR ‘ductal adenocarcinoma’ [tiab] OR ‘Pancreatic Neoplasms’[Mesh])) AND ((pancreaticoduodenectom*[tiab] OR pancreatoduodenectom*[tiab] OR pancreatectom*[tiab] OR duodenopancreatectom*[tiab] OR ((left[tiab] OR distal[tiab]) AND resection*[tiab]) OR Whipple[tiab] OR ppWhipple[tiab] OR dpphr[tiab] OR PPPD[tiab] OR Kausch-Whipple[tiab]) OR (‘Pancreaticoduodenectomy’[Mesh] OR ‘Pancreatectomy’[Mesh]) OR ((pancreas [tiab] OR pancreatic[tiab] OR pancreato*[tiab]) AND (resection* [tiab] OR removal [tiab] OR enucleation* [tiab]))) AND (2010:2021 [dp]).

### Study selection

All randomized trials, observational studies with or without controls, and case series, providing hazard ratios (HRs) for the association of resection margin status and median overall survival (OS) in patients with PDAC who underwent primary resection intent, were included. To limit heterogeneity and associated differences in pathology protocols, the year 2010 as the publication date was chosen as a cutoff for the earliest inclusion date^12^. Exclusion criteria included pancreatic tumours other than PDAC, neoadjuvant chemotherapy, R2 resections, studies not reporting separate HRs, and those not providing detailed information on resection margin definitions. Reviews, meta-analyses, meeting abstracts, letters, comments, editorials, and publications without available full texts were excluded.

Titles and abstracts were independently reviewed by two investigators. Any disagreements were resolved by consensus.

### Data extraction

The two reviewers independently extracted data using a standardized form, which included the following items: title; first author; country; year of publication; journal; study design and period; duration of follow-up; sample size; type of operation; adjuvant chemotherapy regimen; the applied definition of resection margin; examined margins; slicing technique; vascular resections; survival outcomes as median OS; and median time of follow-up.

To account for differences in terminology and to enable cross-comparison, data were extracted based on the distance of tumour cells to the margin. Subsequently, these data were grouped into four categories: R0 (CRM negative (CRM−)), corresponding to 1 mm or more tumour-free margin distance; R0 (CRM positive (CRM+)) with a tumour-free margin of less than 1 mm (classified as R1 in the revised definition)^10^; R1, with tumour cells directly at the resection margin; and R0 direct, with no tumour cells at the resection margin. Rates of margin status were pooled by meta-analysis of proportions.

### Critical appraisal

The risk of bias and the quality of studies was assessed using the Quality in Prognosis Studies (QUIPS) tool^17^. The six respective domains, ‘participation’, ‘attrition’, ‘prognostic factor measurement’, ‘confounding measurement and account’, ‘outcome measurement’, and ‘analysis and reporting’ were graded as low risk, moderate risk, or high risk of bias for each study. Funnel plotting was performed to explore potential bias if more than 10 trials were available. Egger’s test was performed in the case of funnel plot asymmetry^18^.

### Data handling and statistical analysis

The following comparisons between the four groups were performed:

- R1 *versus* R0 direct from on uni- (UV) and multivariable (MV) data
- R1 *versus* R0 (CRM+)
- R1 *versus* R0 (CRM−)
- R0 (CRM−) *versus* R0 (CRM+) from UV and MV data
- Given the small number of studies reporting HRs comparing R0 (CRM−) and R0 (CRM+) separately, and that R0 (CRM+) and R1 are frequently considered together or not fully diversified in some studies, these latter categories were combined: R0 (CRM+) with R1 *versus* R0 (CRM−) from UV and MV data.

Additionally, R0 (CRM+) with R1 was compared to R0 (CRM−) from studies reporting only UV data or MV data, respectively. A subgroup analysis, including all studies reporting HR for pancreatic head tumours, was performed. Data handling was the same as for the whole cohort.

Meta-analyses were carried out with R programming language^19^. Forest plots were generated. A random-effects model was used to account for study heterogeneity. Statistical heterogeneity among the estimated effect of the included studies was evaluated using the *I*^2^ statistic. An *I*^2^ of less than 25 per cent indicated low heterogeneity, while an *I*^2^ of more than 75 per cent a high heterogeneity, with 25 to 75 per cent indicating moderate heterogeneity. HRs were pooled using a random-effects model according to DerSimonian and Laird^20^.

Meta-regression was performed using a mixed-effects model with median follow-up time as a covariate to assess if follow-up time independently influenced results. Meta-regression was limited to group comparisons that included six or more studies.

## Results

### Study characteristics

**Fig. 1:**
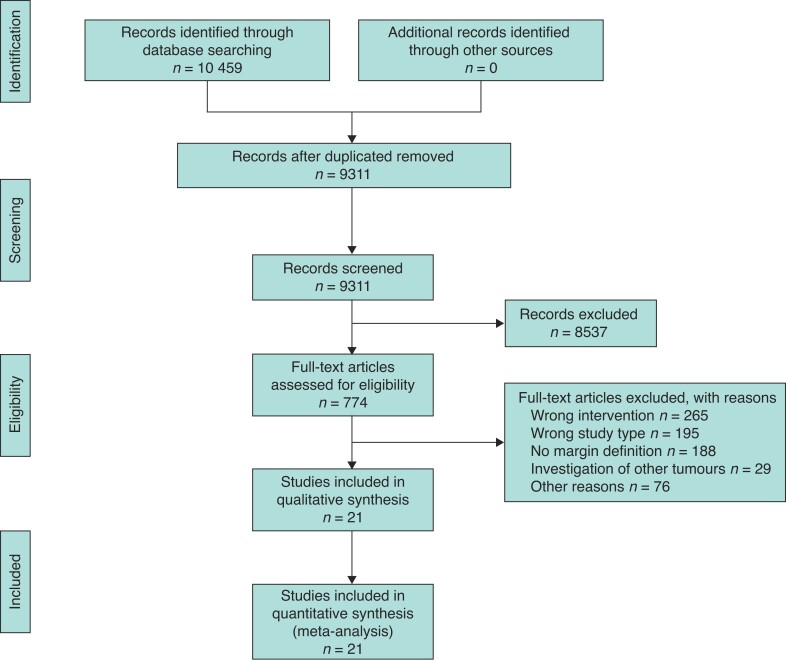
PRISMA flow diagram for inclusion and exclusion of studies

A total of 10 459 articles were potentially eligible. After removing duplicates and screening titles and abstracts, the remaining 774 records were screened in full text. From these, 265 (34.2 per cent) studies were excluded because of other type of intervention or study design (*n* = 195; 25.2 per cent), no margin definitions (*n* = 188; 24.3 per cent), investigation of other tumours (*n* = 29; 3.7 per cent), and other reasons (*n* = 76; 9.8 per cent). A PRISMA flow chart is shown in *Fig. 1*. Finally, 21 studies were included in the qualitative and quantitative synthesis, including 6056 patients (*Table 1*). Of these, 14 were single-centre, while seven were multicentre studies. The majority (*n* = 17) were retrospective series, with four being prospective, two of which were randomized controlled trial. The multicentre trial by Delpero *et al*. prospectively enrolled patients to evaluate the prognostic implications of different resection margin definitions without any randomization procedure^14^, while the study by Jamieson *et al*. mentions a prospective study design without further details^25^.

**Table 1:** Studies included in the final qualitative and quantitative synthesis

First author	Publication year	Number of patients	Procedure	Margin definition*	Follow-up (months)
**Gebauer^11^**	2015	118	PD	Wide	17
**Delpero^14^**	2017	117	PD	Wide	83
**Demir^12^**	2018	254	PD, DP, TP	Wide	47
**Neoptolemos^21^**	2017	730	PD, DP, TP	Wide	43
**Nitta^22^**	2017	117	PD	Wide	47
**Strobel^10^**	2017	561	PD	Wide	29
**van Roessel^23^**	2018	531	PD	Wide	50
**Hank^24^**	2018	455	DP, TP	Wide	33
**Jamieson^25^**	2011	217	PD	Wide	20
**Ghaneh^26^**	2019	1151	PD, DP, TP	Wide	34
**Serenari^27^**	2019	99	n.s.	Wide	n.r.
**Panaro^28^**	2019	79	PD	Wide	30
**Vuarnesson^29^**	2013	188	PD	Wide	45
**Kishi^30^**	2019	500	PD	Narrow	n.r.
**You^31^**	2019	194	PD	Wide	17
**Tummers^32^**	2019	322†	PD, DP, TP	Wide	n.r.
**Li^33^**	2019	124	PD	Narrow	n.r.
**Ocana^34^**	2020	80	PD	Wide	n.r.
**Di Martino^35^**	2020	33	PD	Wide	n.r.
**Pine^36^**	2020	107	PD	Wide	30
**Prochazka^37^**	2020	79	PD, TP	Wide	n.r.

* Wide: 1 mm margin clearance; narrow: direct margin clearance. ^†^Only PD used for association of overall survival and margin status. PD, pancreaticoduodenectomy; DP, distal pancreatic resection; TP, total pancreatectomy; n.s., not specified; n.r., not reported.

On proportional meta-analysis, the R0 (CRM−) rate was 38 per cent (95 per cent confidence interval (c.i.) 29 to 47); the R0 (CRM+) rate was 50 per cent (95 per cent c.i. 40 to 61); the R1 rate was 30 per cent (95 per cent c.i. 20 to 43); and the R0 direct rate was 72 per cent (95 per cent c.i. 69 to 76) (*Fig. S1*).

In total, 4965 patients received either a Whipple pancreaticoduodenectomy (PD) or a pylorus-preserving PD. Of the remaining patients, 440 underwent a total pancreatectomy and 378 a distal pancreatic resection. One study of 99 patients did not specify the type of pancreatic resection performed^27^. The reported median OS in the entire cohort ranged from 12 months for patients with an R1 resection to 62.9 months for those with an R0 (CRM−) resection^22^. The median follow-up time varied between 17 and 83 months but was missing in seven studies (*Table 1*).

### Risk of bias and study heterogeneity

The QUIPS tool showed a moderate risk of bias in most studies, as displayed in the funnel plots (*Fig. S2*). Egger’s test did not reveal significant asymmetry of either funnel plot (*P* = 0.22 and *P* = 0.11, respectively).

### Resection margin and overall survival

#### *R1* versus *R0 direct*

**Fig. 2:**
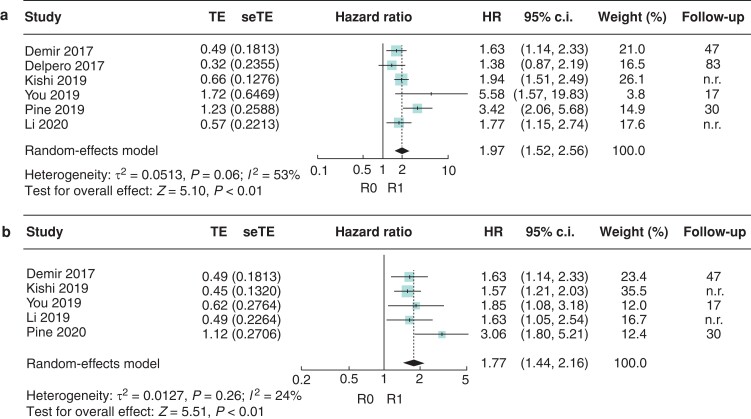
Forest plot of the meta-analysis of median overall survival comparing R1 *versus* R0 direct margin status

Six studies were included in the analysis. Using UV data, the pooled HR for OS comparing R1 with R0 direct resections was 1.97 (95 per cent c.i. 1.52 to 2.56; *I*^2^ = 53 per cent) (*Fig. 2a*). Five of the included studies showed that R0 direct resections were independently associated with an improved OS *versus* R1 resections (HR 1.77, 95 per cent c.i. 1.44 to 2.16; *I*^2^ = 24 per cent) (*Fig. 2b*).

**a** Univariable and **b** multivariable data. TE, estimated treatment effect; seTE, standard error of treatment estimate; n.r., not reported.

#### *R1* versus *R0 (CRM+) and R1* versus *R0 (CRM−)*

**Fig. 3:**
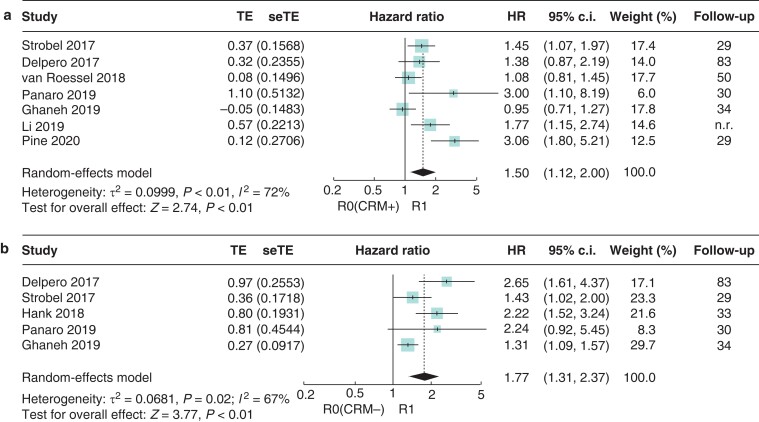
Forest plot of the meta-analysis of median overall survival

Seven studies reported HRs for survival differences between R1 and R0 (CRM+) resections (*Fig. 3a*). Tumour cells directly at the resection margin (R1) were associated with a worse OS than tumour cells less than 1 mm distance from the resection margin (R0 (CRM+); HR 1.50, 95 per cent c.i. 1.12 to 2.00 (*I*^2^ = 72 per cent)). This association was even more pronounced comparing R1 *versus* R0 (CRM−) resections in five eligible studies (HR 1.77, 95 per cent c.i. 1.31 to 2.37; *I*^2^ = 67 per cent) (*Fig. 3b*).

**a** Comparing R0 (CRM+) resections *versus* R1 resections and **b** R0 (CRM−) resections *versus* R1 resections. TE, estimated treatment effect; seTE, standard error of treatment estimate; n.r., not reported.

#### *R0 (CRM−)* versus *R0 (CRM+)*

**Fig. 4:**
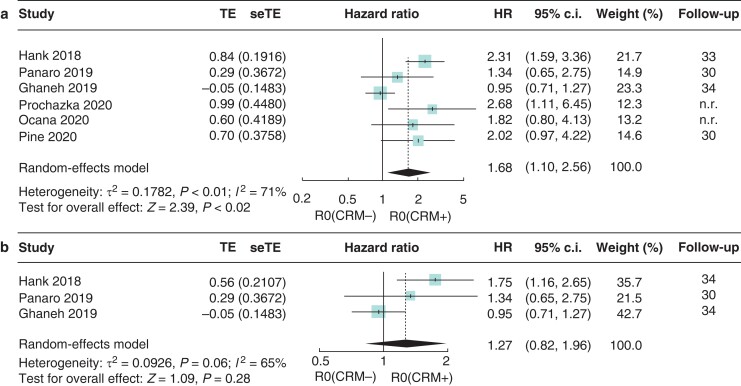
Forest plot of the meta-analysis of median overall survival

Six studies reported separate HRs using UV data (*Fig. 4a*). The revised R0 definition with a margin clearance of 1 mm or more (R0 (CRM−) was associated with an improved OS *versus* R0 (CRM+) resections (HR 1.68, 95 per cent c.i. 1.10 to 2.56). Using MV data (reported in three eligible studies), no significant association was confirmed (HR 1.27, 95 per cent c.i. 0.82 to 1.96) (*Fig. 4b*). Pooled HR from UV and MV data had moderate study heterogeneity(*I*^2^ = 71 per cent and *I*^2^ = 65 per cent, respectively).

Comparing R0 (CRM−) resections *versus* R0 (CRM+) resections using **a** univariable and **b** multivariable data. TE, estimated treatment effect; seTE, standard error of treatment estimate; n.r., not reported.

#### *R0 (CRM−)* versus *R0 (CRM+) and R1*

**Fig. 5:**
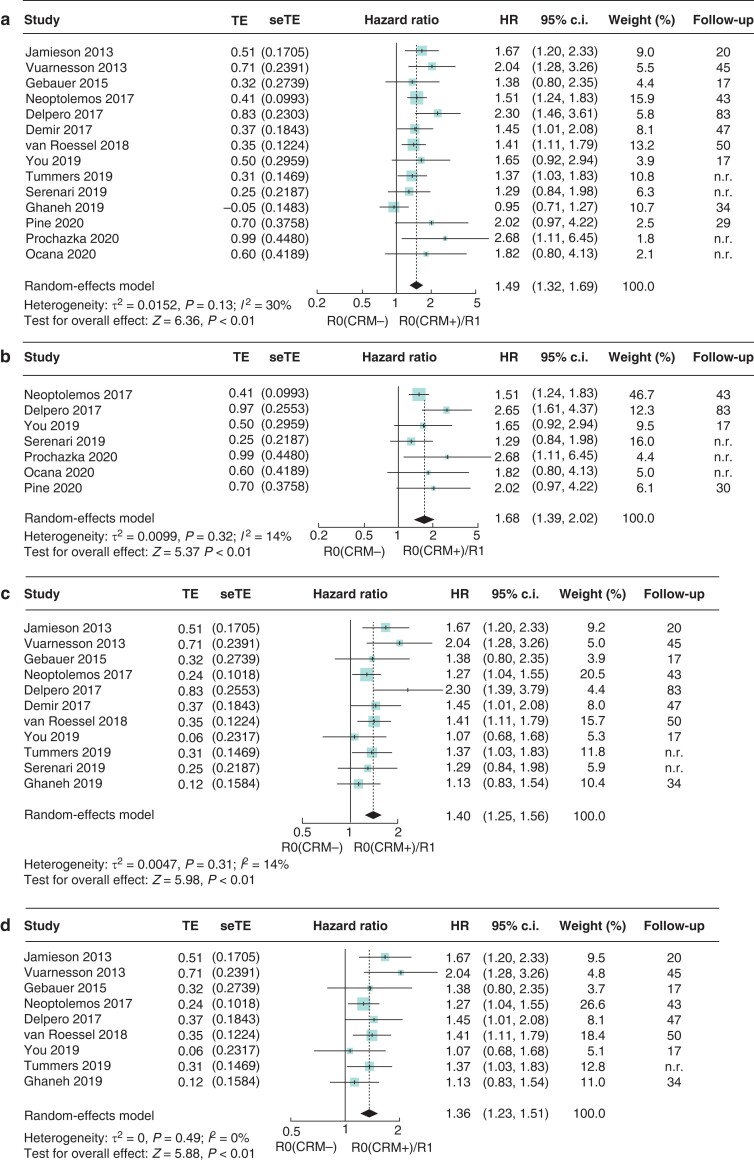
Forest plot of the meta-analysis of median overall survival comparing R0 (CRM−) *versus* the combined category: R0 (CRM+) with R1 resections

**Fig. 6:**
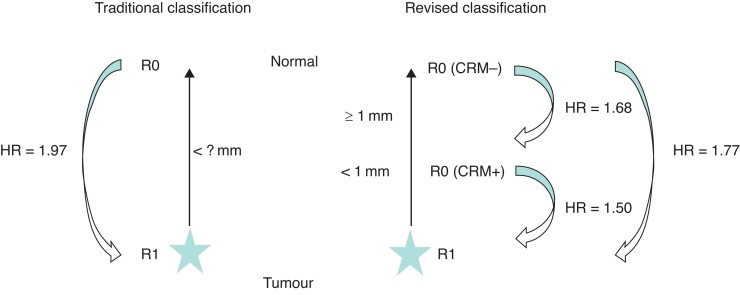
Summary chart comparing resection margin status definitions and associated hazard ratios using univariable data

R0 (CRM−) resections showed a statistically significant survival benefit compared with the combined category: R0 (CRM+) with R1 resections (HR 1.49, 95 per cent c.i. 1.32 to 1.69; *I*^2^ = 30 per cent) (*Fig. 5a*). These results were confirmed when the UV (HR 1.68, 95 per cent c.i. 1.39 to 2.02; *I*^2^ = 14 per cent) (*Fig. 5b*) and MV data (HR 1.40, 95 per cent c.i. 1.25 to 1.56; *I*^2^ = 14 per cent) were considered separately (*Fig. 5c*). Studies reporting HR exclusively from MV analysis confirmed that R0 (CRM−) was significantly associated with an improved median OS (HR 1.36, 95 per cent c.i. 1.23 to 1.51; *I*^2^ = 0 per cent) (*Fig. 5d*). A summary chart comparing the two resection margin status definitions is provided in *Fig. 6*.

**a** Studies reporting univariable hazard ratios and **b** studies reporting only univariable data. **c** Studies reporting hazard ratios from multivariable data and **d** studies reporting hazard ratios only from multivariable data. TE, estimated treatment effect; seTE, standard error of treatment estimate; n.r., not reported.

### Meta-regression analysis

Meta-regression analysis revealed that a median follow-up time of 83 months had an independent effect on results of R0 (CRM−) and R0 (CRM+) using MV data and between R0 direct and R1 using UV data (*P* = 0.03 and *P* = 0.04, respectively). In the meta-regression analyses of other subgroup analyses, follow-up time had no independent effect on median overall survival (*Table 1*).

### Subgroup analysis of pancreatic head tumours

A subgroup analysis of studies reporting separate HRs for PDAC of the pancreatic head was performed. The association between margin status and OS mimicked the results obtained by including all types of resections (*Figs S3 to S5*).

## Discussion

This systematic review and meta-analysis provided evidence that a circumferential margin of resection of 1 mm or more (according to the revised R0 definition) was associated with improved survival compared to less than 1 mm in patients with PDAC after primary pancreatic resections. This survival benefit was evident from UV but not MV analysis, probably reflecting the limited number of studies available.

Because of the small number of eligible studies, the groups with positive margins, namely R0 (CRM+; margin clearance less than 1 mm) and R1 indicating direct margin involvement, were combined. A significant and independent survival benefit for R0 (CRM−) resections (1 mm tumour-free margin) was identified, irrespective of the R1 definition used.

Variations in specimen processing could also explain the lack of R0 (CRM−) confirmation as an independent predictor for longer OS than R0 (CRM+) using MV data. Similarly, the relatively low pooled R0 (CRM+) rate of approximately 58 per cent compared to large single-centre studies with standardized pathology protocols has probably been affected by such variability^2,10^. Pathology protocol differences across various centres are reflected in this meta-analysis. While axial slicing techniques are commonly used in Europe, pathologists in the USA frequently opt for bivalving protocols^5^, and, in some studies, the pathology protocol was not reported^22,30^. Although it is accepted that standardized pathology protocols and resection margin definitions strongly influence positivity rates^38^, the Dutch APOLLO randomized trial, which compared axial slicing and bivalving protocols, failed to detect a significant difference in R1 rates^39^.

Microscopic tumour infiltration occurs mainly at the medial and posterior cut surface, and, in most cases, only one is involved^3,12,13,40–42^. A handful of studies have rigorously addressed which cut surface has the greatest implications for patient outcome, but the number of margins examined and the terminology has varied^5^. Data from the ESPAC-3 trial were analysed to assess the prognostic implications of each cut surface. Positivity of the posterior margin was associated with significantly shorter survival, while a positive anterior cut surface was not associated with reduced survival compared to an overall R0 of 1 mm or more on UV analysis^26^.

Other studies have not detected an association between affected margin locations and survival^32^. The 8th edition of the AJCC Cancer Staging Manual explicitly refers to the uncinate margin for the circumferential assessment, but the others remain unspecified^5,9^. Tummers *et al*. found no significant difference in local recurrence between R1 of less than 1 mm *versus* R0 of 1 mm or more. Once a subgroup analysis was performed, stratifying for lymph node involvement, the R1 groups (R1N0 and R1N1) showed local recurrence significantly earlier^32^.

A recent large institutional series showed that the revised R0 definition was also an independent prognostic determinant in these patients, and an R0 (CRM−) resection may identify a subset with a favourable prognosis and a median OS of 62.4 months^24^.

Recently, novel adjuvant chemotherapy regimens have significantly improved median OS in PDAC patients undergoing upfront surgery^21,43^. Although a separate and stratified analysis is desirable, few studies strictly defined margin status to analyse their prognostic significance given the regimen of adjuvant chemotherapy used. The importance of resection margins after neoadjuvant chemotherapy has recently been addressed, but the results are controversial^44–47^.

A wide R0 margin definition may also have a prognostic relevance as PDAC exhibits a diffuse infiltrative growth pattern^40^. Chang *et al*. identified a distance of 1.5 mm or more as a new cut-off for better outcomes in a cohort of 365 upfront resected patients with disease-specific survival as primary endpoint^42^. Delpero *et al*. showed that clearance of 1.0 mm or less and 1.5 mm or less for at least two positive margins were independent determinants of decreased survival^14^. Although with interesting implications, the limited number of studies addressing the prognostic implication of different tumour cell distances to resection margins prevented such an analysis in this study.

This systematic review has several strengths. The selection was limited to studies published after 2010 as earlier studies frequently did not apply standardized protocols for pathological assessment and adjuvant chemotherapy regimens varied significantly^12^. While this time frame reduced the number of included studies, it strengthened the validity of conclusions. Only studies that provided detailed information on resection margins were included, and all studies that did not report separate HRs for OS in primary resections were excluded.

This study has several limitations. Most of the included studies had a retrospective design, associated with inherent flaws, including selection bias. Owing to the strict inclusion criteria, the number of appropriate studies identified was relatively small, preventing subgroup analysis.

In summary, the revised R status definition is valid and shows a favourable prognosis compared to R0 (CRM+) and R1. A key limitation of a systematic review is the quality of the original studies contained within. This study used the QUIPS tool to assess study quality and risk fo bias. While the risk of bias was rated as moderate and Egger’s test did not reveal significant asymmetry, this systematic review highlights that most studies lack essential information on margin definitions and applied pathology protocols. Furthermore, most studies were of retrospective design and therefore of limited quality. This limits the conclusions of this systematic review and meta-analysis. Detailed information on pathological specimen processing and standardised reporting is necessary for further progress in PDAC treatments and facilitates comparisons between studies and institutions. The implications of the revised R status after neoadjuvant treatment and pancreatic resections other than PD require further investigation.

## Supplementary Material

zrac010_Supplementary_Data

## Data Availability

Data relating to the results of this study, including search strategy, are available upon request by contacting the corresponding author. Search strategies for Web of Science and CENTRAL are available upon request.

## References

[1] Rahib L, Smith BD, Aizenberg R, Rosenzweig AB, Fleshman JM, Matrisian LM. Projecting cancer incidence and deaths to 2030: the unexpected burden of thyroid, liver, and pancreas cancers in the United States. Cancer Res 2014;74:2913–2921 24840647 10.1158/0008-5472.CAN-14-0155

[2] Esposito I, Kleeff J, Bergmann F, Reiser C, Herpel E, Friess H et al Most pancreatic cancer resections are R1 resections. Ann Surg Oncol 2008;15:1651–1660 18351300 10.1245/s10434-008-9839-8

[3] Schlitter AM, Esposito I. Definition of microscopic tumor clearance (r0) in pancreatic cancer resections. Cancers 2010;2:2001–2010 24281214 10.3390/cancers2042001 PMC3840457

[4] Bockhorn M, Uzunoglu FG, Adham M, Imrie C, Milicevic M, Sandberg AA et al Borderline resectable pancreatic cancer: a consensus statement by the international study group of pancreatic surgery (ISGPS). Surgery 2014;155:977–988 24856119 10.1016/j.surg.2014.02.001

[5] Shi J, Basturk O. Whipple grossing in the era of new staging: should we standardise? Diagnostics 2019;9:132 31569496 10.3390/diagnostics9040132 PMC6963989

[6] Campbell F, Bennett M, Foulis A. Minimum Dataset for Histopathological Reporting of Pancreatic, Ampulla of Vater and Bile Duct Carcinoma. Vol. v3. London: Royal College of Pathologists, 2002, 1–63

[7] Campbell F, Smith RA, Whelan P, Sutton R, Raraty M, Neoptolemos JP et al Classification of R1 resections for pancreatic cancer: the prognostic relevance of tumour involvement within 1mm of a resection margin. Histopathology 2009;55:277–283 19723142 10.1111/j.1365-2559.2009.03376.x

[8] Verbeke CS, Leitch D, Menon KV, McMahon MJ, Guillou PJ, Anthoney A. Redefining the R1 resection in pancreatic cancer. Br J Surg 2006;93:1232–1237 16804874 10.1002/bjs.5397

[9] Amin MB, American Joint Committee on Cancer, American Cancer Society. AJCC Cancer Staging Manual. Chicago, IL: American Joint Committee on Cancer, Springer, 2017

[10] Strobel O, Hank T, Hinz U, Bergmann F, Schneider L, Springfeld C et al Pancreatic cancer surgery: the new R-status counts. Ann Surg 2017;265:565–573 27918310 10.1097/SLA.0000000000001731

[11] Gebauer F, Tachezy M, Vashist YK, Marx AH, Yekebas E, Izbicki JR et al Resection margin clearance in pancreatic cancer after implementation of the Leeds pathology protocol (LEEPP): clinically relevant or just academic? World J Surg 2015;39:493–499 25270344 10.1007/s00268-014-2808-4

[12] Demir IE, Jäger C, Schlitter AM, Konukiewitz B, Stecher L, Schorn S et al R0 versus R1 resection matters after pancreaticoduodenectomy, and less after distal or total pancreatectomy for pancreatic cancer. Ann Surg 2018;268:1058–1068 28692477 10.1097/SLA.0000000000002345

[13] Chandrasegaram MD, Goldstein D, Simes J, Gebski V, Kench JG, Gill AJ et al Meta-analysis of radical resection rates and margin assessment in pancreatic cancer. Br J Surg 2015;102:1459–1472 26350029 10.1002/bjs.9892

[14] Delpero JR, Jeune F, Bachellier P, Regenet N, Le Treut YP, Paye F et al Prognostic value of resection margin involvement after pancreaticoduodenectomy for ductal adenocarcinoma: updates from a French prospective multicenter study. Ann Surg 2017;266:787–796 28953554 10.1097/SLA.0000000000002432

[15] Moher D, Liberati A, Tetzlaff J, Altman DG, Group P. Preferred reporting items for systematic reviews and meta-analyses: the PRISMA statement. PLoS Med 2009;6:e1000097 19621072 10.1371/journal.pmed.1000097 PMC2707599

[16] Goossen K, Tenckhoff S, Probst P, Grummich K, Mihaljevic AL, Buchler MW et al Optimal literature search for systematic reviews in surgery. Langenbecks Arch Surg 2018;403:119–129 29209758 10.1007/s00423-017-1646-x

[17] Hayden JA, van der Windt DA, Cartwright JL, Cote P, Bombardier C. Assessing bias in studies of prognostic factors. Ann Intern Med 2013;158:280–286 23420236 10.7326/0003-4819-158-4-201302190-00009

[18] Egger M, Davey Smith G, Schneider M, Minder C. Bias in meta-analysis detected by a simple, graphical test. BMJ 1997;315:629–634 9310563 10.1136/bmj.315.7109.629 PMC2127453

[19] Ihaka R, Gentleman R. R: a language for data analysis and graphics. J Comput Graph Stat 1996;5:299–314

[20] DerSimonian R, Laird N. Meta-analysis in clinical trials. Control Clin Trials 1986;7:177–188 3802833 10.1016/0197-2456(86)90046-2

[21] Neoptolemos JP, Palmer DH, Ghaneh P, Psarelli EE, Valle JW, Halloran CM et al Comparison of adjuvant gemcitabine and capecitabine with gemcitabine monotherapy in patients with resected pancreatic cancer (ESPAC-4): a multicentre, open-label, randomised, phase 3 trial. Lancet 2017;389:1011–1024 28129987 10.1016/S0140-6736(16)32409-6

[22] Nitta T, Nakamura T, Mitsuhashi T, Asano T, Okamura K, Tsuchikawa T et al The impact of margin status determined by the one-millimeter rule on tumor recurrence and survival following pancreaticoduodenectomy for pancreatic ductal adenocarcinoma. Surg Today 2017;47:490–497 27677294 10.1007/s00595-016-1420-7

[23] van Roessel S, Kasumova GG, Tabatabaie O, Ng SC, van Rijssen LB, Verheij J et al Pathological margin clearance and survival after pancreaticoduodenectomy in a US and European pancreatic center. Ann Surg Oncol 2018;25:1760–1767 29651577 10.1245/s10434-018-6467-9 PMC5928169

[24] Hank T, Hinz U, Tarantino I, Kaiser J, Niesen W, Bergmann F et al Validation of at least 1 mm as cut-off for resection margins for pancreatic adenocarcinoma of the body and tail. Br J Surg 2018;105:1171–1181 29738626 10.1002/bjs.10842

[25] Jamieson NB, Denley SM, Logue J, MacKenzie DJ, Foulis AK, Dickson EJ et al A prospective comparison of the prognostic value of tumor- and patient-related factors in patients undergoing potentially curative surgery for pancreatic ductal adenocarcinoma. Ann Surg Oncol 2011;18:2318–2328 21267785 10.1245/s10434-011-1560-3

[26] Ghaneh P, Kleeff J, Halloran CM, Raraty M, Jackson R, Melling J et al The impact of positive resection margins on survival and recurrence following resection and adjuvant chemotherapy for pancreatic ductal adenocarcinoma. Ann Surg 2019;269:520–529 29068800 10.1097/SLA.0000000000002557

[27] Serenari M, Ercolani G, Cucchetti A, Zanello M, Prosperi E, Fallani G et al The impact of extent of pancreatic and venous resection on survival for patients with pancreatic cancer. Hepatobiliary Pancreat Dis Int 2019;18:389–394 31230959 10.1016/j.hbpd.2019.06.004

[28] Panaro F, Kellil T, Vendrell J, Sega V, Souche R, Piardi T et al Microvascular invasion is a major prognostic factor after pancreatico-duodenectomy for adenocarcinoma. J Surg Oncol 2019;120:483–493 31197842 10.1002/jso.25580

[29] Vuarnesson H, Lupinacci RM, Semoun O, Svrcek M, Julié C, Balladur P et al Number of examined lymph nodes and nodal status assessment in pancreaticoduodenectomy for pancreatic adenocarcinoma. Eur J Surg Oncol 2013;39:1116–1121 23948704 10.1016/j.ejso.2013.07.089

[30] Kishi Y, Nara S, Esaki M, Hiraoka N, Shimada K. Feasibility of resecting the portal vein only when necessary during pancreatoduodenectomy for pancreatic cancer. BJS Open 2019;3:327–335 31183449 10.1002/bjs5.50130 PMC6551409

[31] You Y, Choi DW, Heo JS, Han IW, Choi SH, Jang KT et al Clinical significance of revised microscopic positive resection margin status in ductal adenocarcinoma of pancreatic head. Ann Surg Treat Res 2019;96:19–26 30603630 10.4174/astr.2019.96.1.19 PMC6306502

[32] Tummers WS, Groen JV, Sibinga Mulder BG, Farina-Sarasqueta A, Morreau J, Putter H et al Impact of resection margin status on recurrence and survival in pancreatic cancer surgery. Br J Surg 2019;106:1055–1065 30883699 10.1002/bjs.11115 PMC6617755

[33] Li CG, Zhou ZP, Tan XL, Gao YX, Wang ZZ, Liu Q et al Impact of resection margins on long-term survival after pancreaticoduodenectomy for pancreatic head carcinoma. World J Clin Cases 2019;7:4186–4195 31911899 10.12998/wjcc.v7.i24.4186 PMC6940347

[34] Ocaña J, Sanjuanbenito A, García A, Molina JM, Lisa E, Mendía E et al Relevance of positive resection margins in ductal pancreatic adenocarcinoma and prognostic factors. Cir Esp 2020;98:85–91 31395275 10.1016/j.ciresp.2019.06.017

[35] Di Martino M, Ielpo B, de Nova JLM, Muñoz EA, Santamaria C, Diago V et al Lymph node ratio, perineural invasion and R1 resection as independent prognostic factors in pancreatic adenocarcinoma: a retrospective cohort study. Surg Technol Int 2020;36:82–88 32190897

[36] Pine JK, Haugk B, Robinson SM, Darne A, Wilson C, Sen G et al Prospective assessment of resection margin status following pancreaticoduodenectomy for pancreatic ductal adenocarcinoma after standardisation of margin definitions. Pancreatology 2020;20:537–544 31996296 10.1016/j.pan.2020.01.004

[37] Prochazka V, Hlavsa J, Kunovsky L, Farkasova M, Potrusil M, Andrasina T et al Correlation of survival length after pancreaticoduodenectomy for pancreatic head adenocarcinoma depending on tumor characteristics detected by means of computed tomography and resection margins status. Neoplasma 2020;67:1319–1328 32614234 10.4149/neo_2020_190923N955

[38] Esposito I, Konukiewitz B, Schlitter AM, Kloppel G. Pathology of pancreatic ductal adenocarcinoma: facts, challenges and future developments. World J Gastroenterol 2014;20:13833–13841 25320520 10.3748/wjg.v20.i38.13833 PMC4194566

[39] van Roessel S, Soer EC, van Dieren S, Koens L, van Velthuysen MLF, Doukas M, et al Axial slicing versus bivalving in the pathological examination of pancreatoduodenectomy specimens (APOLLO): a multicentre randomised controlled trial. HPB (Oxford) 2021;23:1349–1359 33563546 10.1016/j.hpb.2021.01.005

[40] Verbeke CS . Resection margins and R1 rates in pancreatic cancer–are we there yet? Histopathology 2008;52:787–796 18081813 10.1111/j.1365-2559.2007.02935.x

[41] Takahashi D, Kojima M, Sugimoto M, Kobayashi S, Takahashi S, Konishi M et al Pathologic evaluation of surgical margins in pancreatic cancer specimens using color coding with tissue marking dyes. Pancreas 2018;47:830–836 29975353 10.1097/MPA.0000000000001106

[42] Chang DK, Johns AL, Merrett ND, Gill AJ, Colvin EK, Scarlett CJ et al Margin clearance and outcome in resected pancreatic cancer. J Clin Oncol 2009;27:2855–2862 19398572 10.1200/JCO.2008.20.5104

[43] Neoptolemos JP, Stocken DD, Friess H, Bassi C, Dunn JA, Hickey H et al A randomised trial of chemoradiotherapy and chemotherapy after resection of pancreatic cancer. N Engl J Med 2004;350:1200–1210 15028824 10.1056/NEJMoa032295

[44] Kaltenmeier C, Nassour I, Hoehn RS, Khan S, Althans A, Geller DA et al Impact of resection margin status in patients with pancreatic cancer: a national cohort study. J Gastrointest Surg 2020;25:2307–2316 33269460 10.1007/s11605-020-04870-6 PMC8169716

[45] de Geus SWL, Kasumova GG, Sachs TE, Ng SC, Kent TS, Moser AJ, et al Neoadjuvant therapy affects margins and margins affect all: perioperative and survival outcomes in resected pancreatic adenocarcinoma. HPB (Oxford) 2018;20:573–581 29426635 10.1016/j.hpb.2017.12.004

[46] Lof S, Korrel M, van Hilst J, Alseidi A, Balzano G, Boggi U et al Impact of neoadjuvant therapy in resected pancreatic ductal adenocarcinoma of the pancreatic body or tail on surgical and oncological outcome: a propensity-score matched multicenter study. Ann Surg Oncol 2020;27:1986–1996 31848815 10.1245/s10434-019-08137-6 PMC7210228

[47] Maeda S, Moore AM, Yohanathan L, Hata T, Truty MJ, Smoot RL et al Impact of resection margin status on survival in pancreatic cancer patients after neoadjuvant treatment and pancreatoduodenectomy. Surgery 2020;167:803–811 31992444 10.1016/j.surg.2019.12.008

