# Supplementary figures and images for: Prognostic relevance of the revised R status definition in pancreatic cancer: meta-analysis
Source: BJS Open. 2022 Mar 18;6(2):zrac010. doi: 10.1093/bjsopen/zrac010 (PMC8931487; doi:10.1093/bjsopen/zrac010)

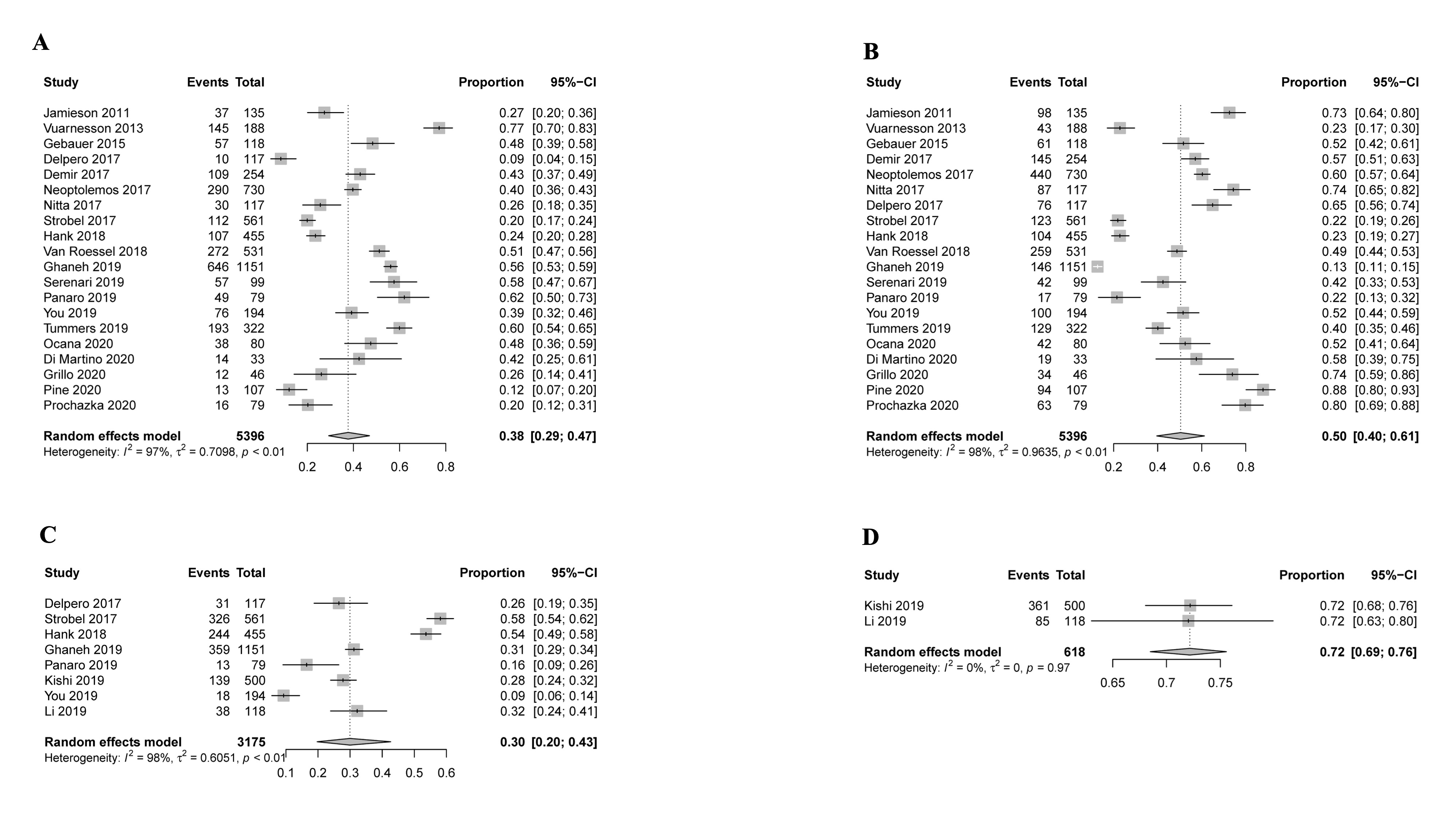

Supplement: zrac010_Supplementary_Data [file zrac010_supplementary_data.zip › Supplementary_Figure_1.jpg]

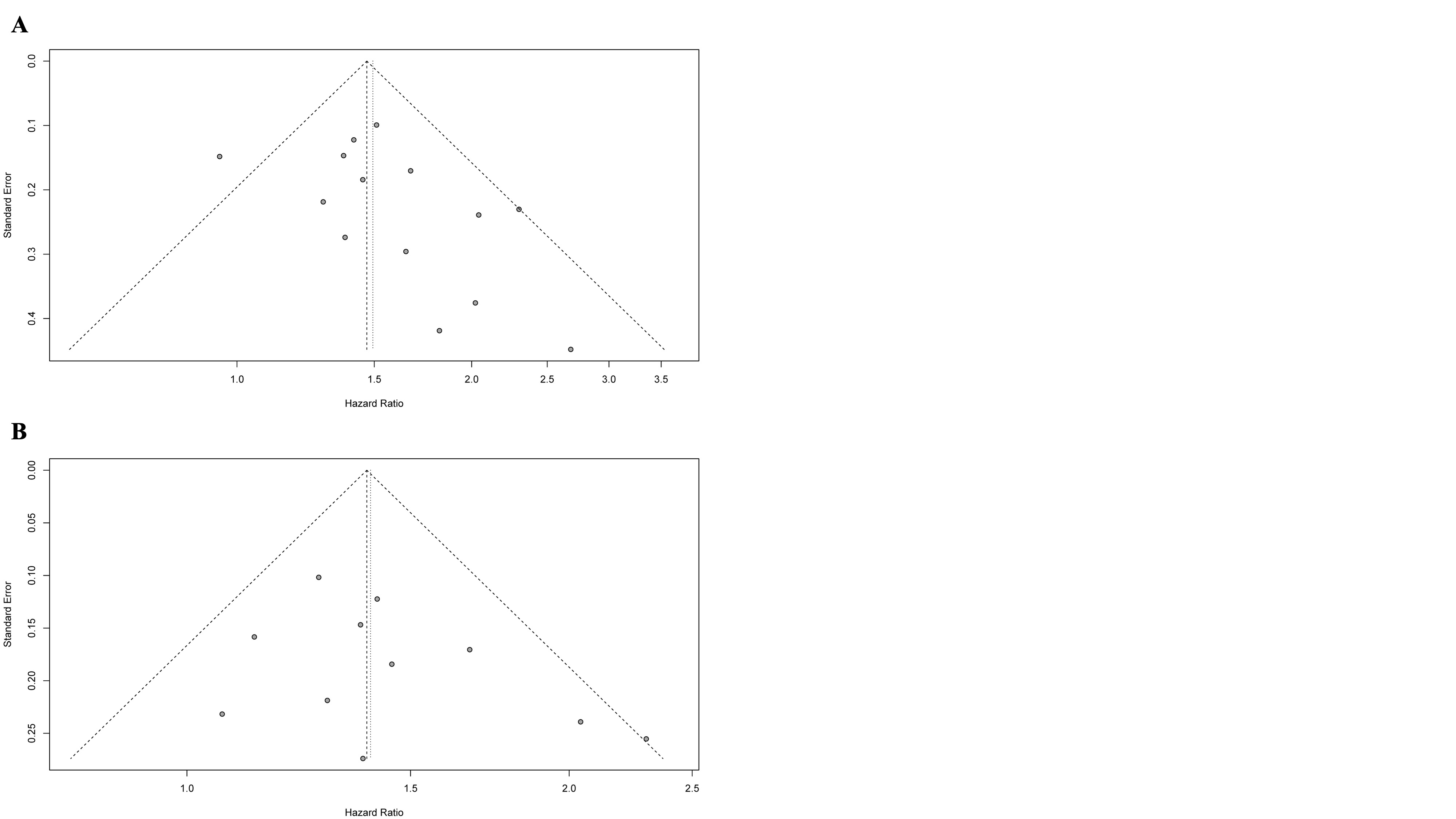

Supplement: zrac010_Supplementary_Data [file zrac010_supplementary_data.zip › Supplementary_Figure_2.jpg]

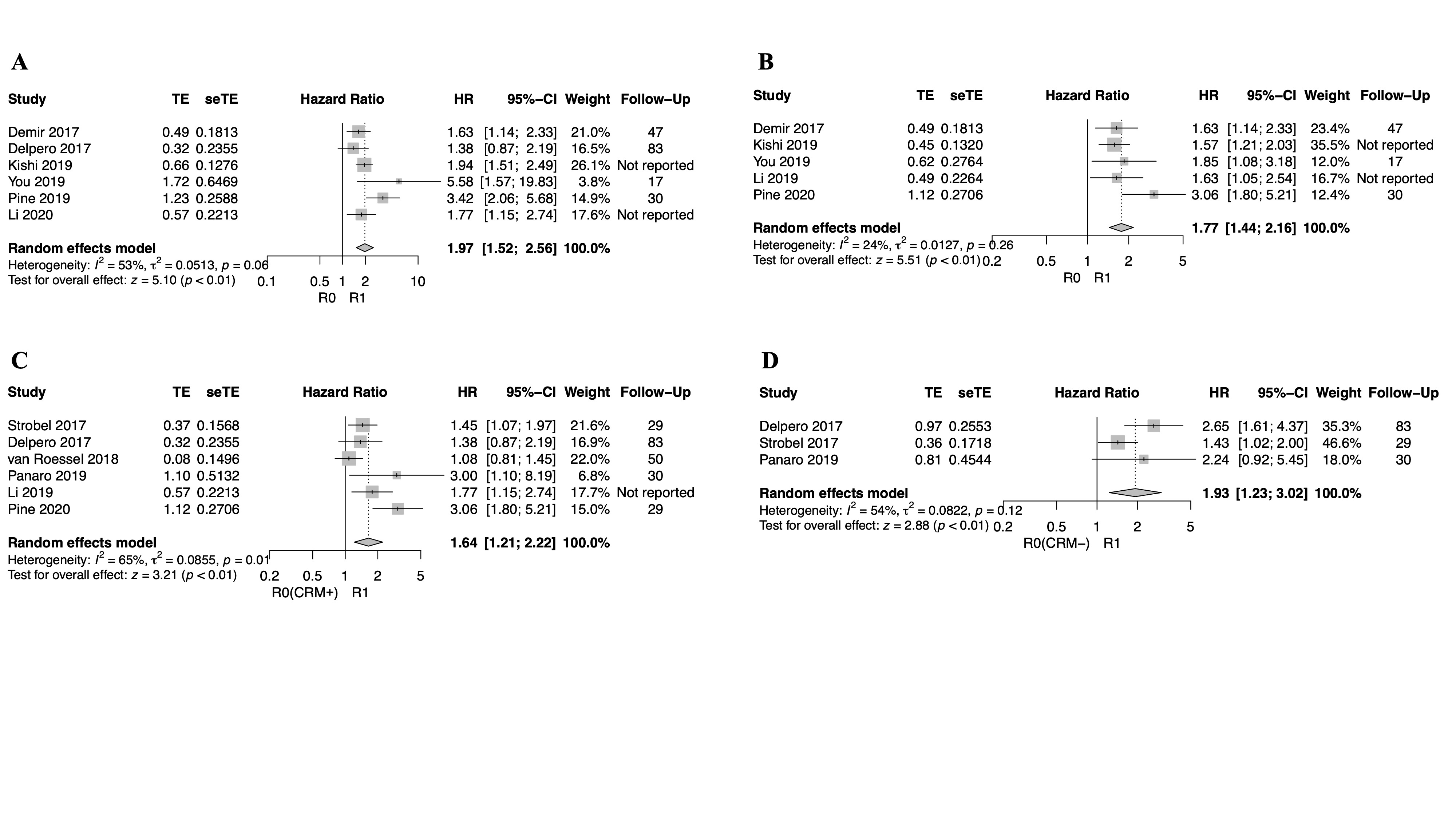

Supplement: zrac010_Supplementary_Data [file zrac010_supplementary_data.zip › Supplementary_Figure_3.jpg]

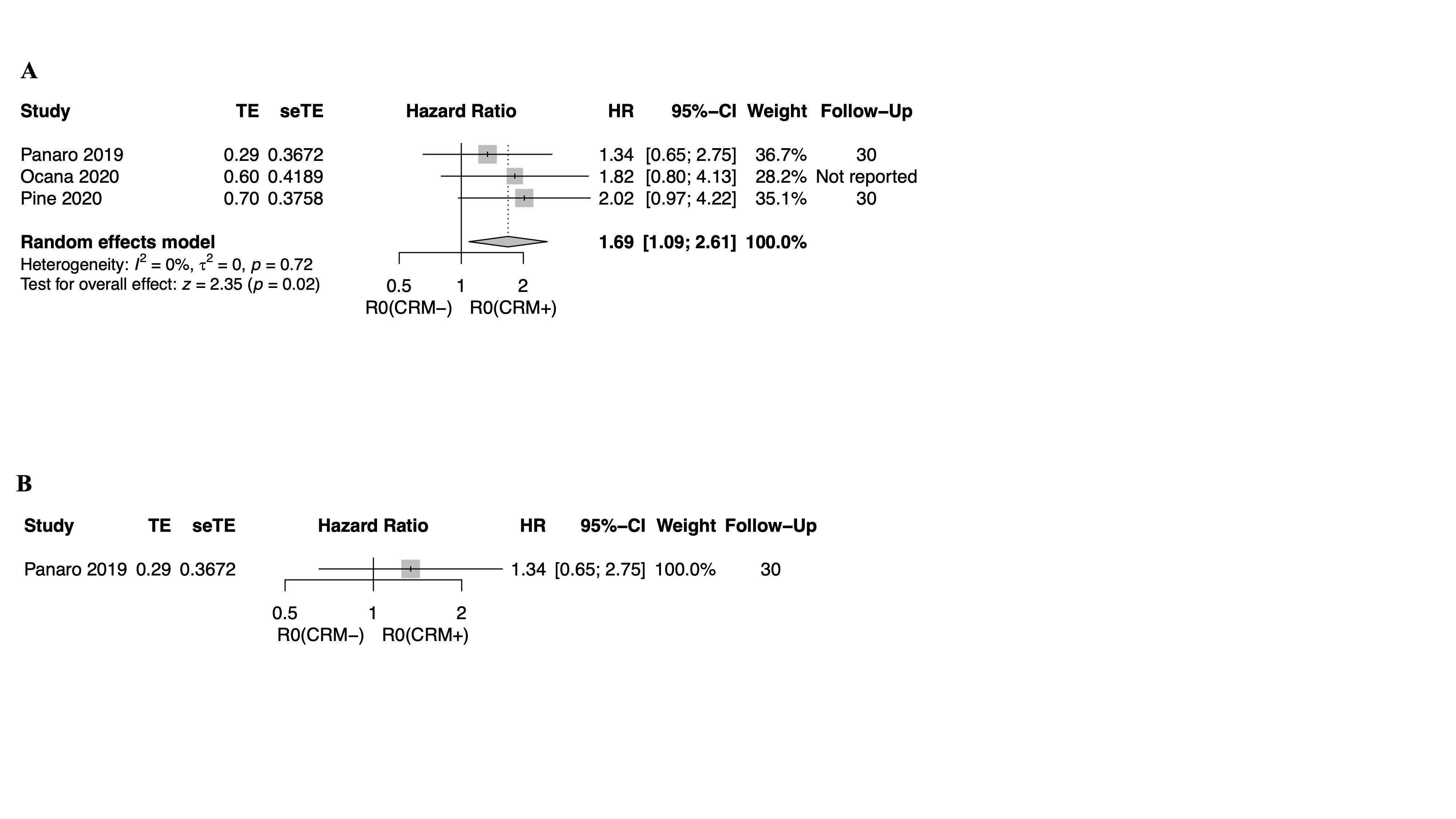

Supplement: zrac010_Supplementary_Data [file zrac010_supplementary_data.zip › Supplementary_Figure_4.jpg]

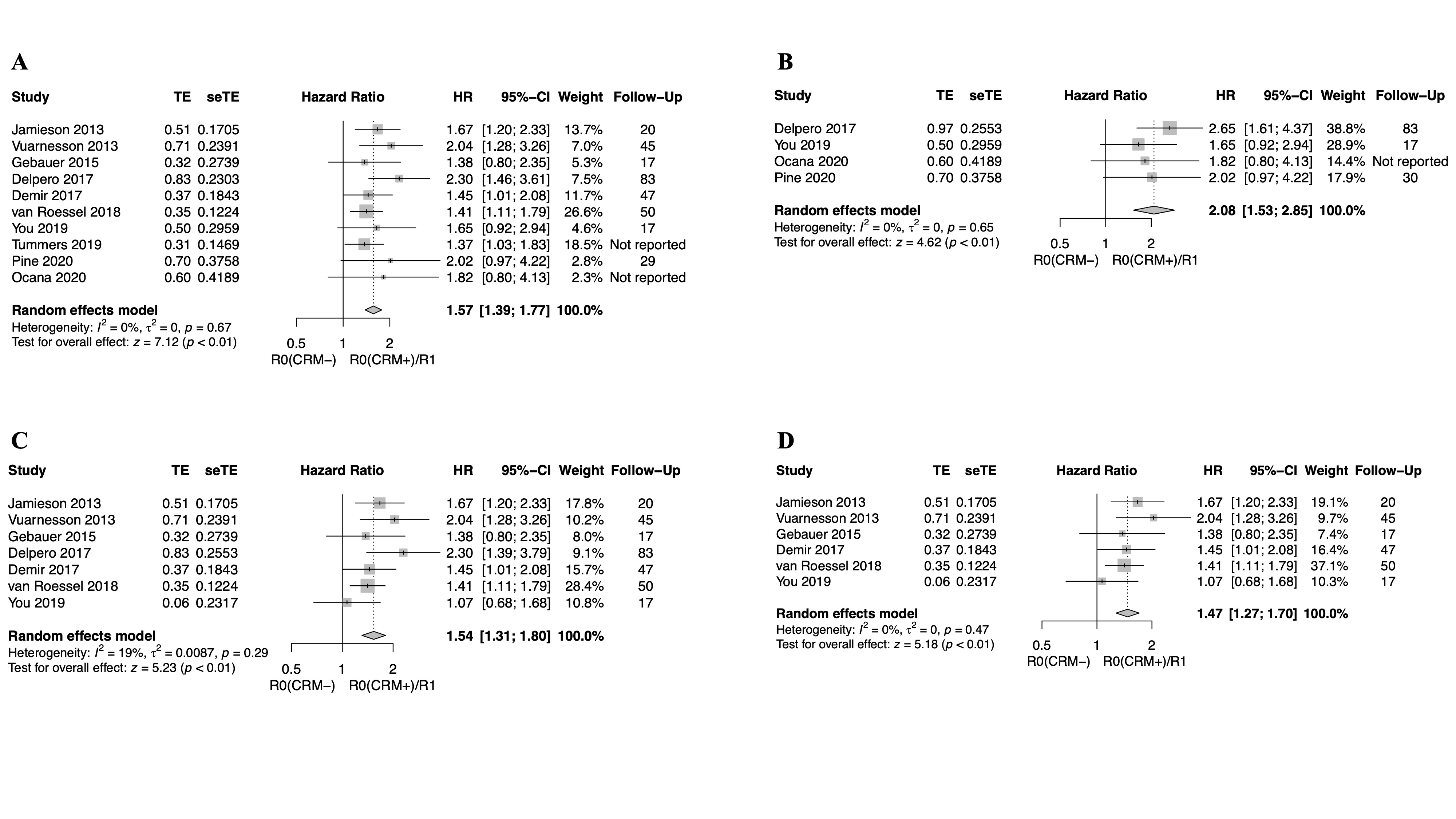

Supplement: zrac010_Supplementary_Data [file zrac010_supplementary_data.zip › Supplementary_Figure_5.jpg]
